# Tree Diversity, Floristic Composition, and Forest Structure in Three Permanent Montane Forest Plots in Northern Peru

**DOI:** 10.3390/biology15191679

**Published:** 2026-09-22

**Authors:** Rosalynn Yohanna Rivera López, Carlos Reynel Rodriguez, Jürgen Homeier, Helge Walentowski, Jaris Veneros

**Affiliations:** 1 Programa Doctoral en Ciencias para el Desarrollo Sustentable, Escuela de Posgrado, Facultad de Ingeniería Zootecnista, Agronegocios, Biotecnología y Ciencia de Datos, Universidad Nacional Toribio Rodríguez de Mendoza de Amazonas, Chachapoyas 01001, Peru; 2 Instituto de Investigación para el Desarrollo Sustentable de Ceja de Selva (INDES-CES), Universidad Nacional Toribio Rodríguez de Mendoza de Amazonas, Chachapoyas 01001, Peru; jaris.veneros@untrm.edu.pe; 3 Herbario Forestal MOL-F, Facultad de Ciencias Forestales, Universidad Nacional Agraria La Molina (UNALM), Av. La Molina s/n, La Molina, Lima 15024, Peru; reynel@lamolina.edu.pe; 4 Faculty of Resource Management, HAWK University of Applied Sciences and Arts, 37075 Göttingen, Germany; juergen.homeier@hawk.de (J.H.); helge.walentowski@hawk.de (H.W.)

**Keywords:** Andean forests, community composition, elevational gradient, forest conservation, floristic turnover, tropical Andes

## Abstract

Tropical montane forests provide essential ecosystem services and are of considerable ecological importance. However, knowledge gaps remain regarding how tree diversity, floristic composition, and forest structure vary among permanent plots across the sampled elevational range (2169–2519 m a.s.l.). We established three independent 1 ha permanent forest plots, with a single plot representing each sampled elevation (2169, 2347, and 2519 m a.s.l.). Because each sampled elevation was represented by a single plot, this design does not provide replication for formal statistical testing of elevational trends; therefore, differences among the three plots were interpreted descriptively. All trees with DBH ≥ 10 cm were inventoried to characterize tree diversity, floristic composition, and forest structure. Diversity was estimated using Hill numbers, and floristic differentiation was assessed using non-metric multidimensional scaling (NMDS). A total of 2336 individuals belonging to 46 families, 75 genera, and 147 species were recorded. Alpha diversity differed among plots, with values ranging from 49 to 67 species (q = 0), 17.2 to 24.6 (q = 1), and 10.7 to 13.6 (q = 2). P-OC1 showed the highest observed species richness and floristic diversity among the three plots. Lauraceae was the most diverse family, while the ecological importance of dominant families, as indicated by FIVI, varied among plots. Comparison with nine additional permanent plots from montane forests of northern, central, and southern Peru revealed floristic differentiation among plots in the NMDS ordination (stress = 0.068). Neither elevation (R^2^ = 0.280, *p* = 0.223) nor species richness (R^2^ = 0.044, *p* = 0.826) was significantly associated with the ordination. This study establishes an ecological baseline for the three permanent plots evaluated and expands the available information on the diversity, composition, and structure of montane forests in northern Peru.

## Simple Summary

Montane forests in northern Peru remain poorly represented in studies based on permanent plots. In this study, we established three 1 ha plots located at 2169, 2347 and 2519 m a.s.l., where we recorded 2336 trees and 147 species. Tree diversity and floristic composition varied among plots. Comparison with other montane forests in Peru showed that elevation contributes to differences in floristic composition, although it does not explain all the observed variation. The information obtained expands our knowledge of these forests in Amazonas and establishes a baseline for their monitoring and conservation.

## 1. Introduction

Tropical montane forests (TMFs) are highly diverse and heterogeneous ecosystems, harboring high levels of floristic richness, endemism, and functional diversity across complex environmental gradients [1,2,3]. In the tropical Andes, these forests play a fundamental role in biodiversity conservation, hydrological regulation, and the maintenance of ecological processes, while also providing important ecosystem services to human populations [4,5,6]. In northern Peru, many montane forest remnants are conserved within communal lands, making local communities key stakeholders in biodiversity conservation and long-term forest management. However, despite their ecological and environmental importance, knowledge of patterns of diversity, floristic composition, and forest structure remains limited in many Andean regions, particularly in the montane forests of northern Peru [7,8].

Environmental variation associated with elevation is an important source of ecological differentiation in tropical montane forests, influencing tree diversity, floristic composition, and forest structure [2,9,10]. Changes in temperature, moisture, soils, and topography along mountain landscapes can contribute to species turnover and shifts in the abundance and dominance of tree lineages, while structural attributes such as stem density, basal area, diameter distribution, and vertical stratification may also vary among forest stands [9,10,11]. However, these patterns are not uniform across regions or even among forests occurring at similar elevations, indicating that local environmental conditions, disturbance history, and biogeographic factors may interact with elevation in shaping montane forest communities [4,10,12]. Therefore, assessing diversity, floristic composition, and forest structure together provides a more complete characterization of ecological variation among montane forests without attributing the observed differences to elevation alone.

Floristic composition in tropical montane forests often varies across elevational ranges as species, genera, and families respond differently to environmental conditions associated with elevation. Changes in temperature, water availability, cloud immersion, soil properties, and other environmental filters can influence species establishment and performance, resulting in differences in taxonomic composition among forest stands [9,10,11,13]. In the tropical Andes, such variation has been reflected in differences in the abundance and dominance of families such as Lauraceae, Melastomataceae, Cunoniaceae, and Clusiaceae [9,10,14], while shifts in community composition along environmental gradients may also reflect contrasting functional strategies and ecological tolerances among tree taxa [15,16]. However, forests occurring at similar elevations can also differ markedly in composition because elevation interacts with local environmental heterogeneity, disturbance history, and biogeographic factors [2,4,12,16].

The montane forests of northern Peru harbor a diverse flora and numerous taxa of conservation concern, including endemic species and species classified under different threat categories at both national and international levels, such as Critically Endangered (CR), Endangered (EN), Vulnerable (VU), and Near Threatened (NT), according to the IUCN Red List and current Peruvian legislation [7,8,17,18,19,20,21]. Therefore, assessing floristic composition and conservation status provides an opportunity to identify species with restricted distributions and document new regional records relevant to the Andean flora [22,23,24,25]. Likewise, integrating taxonomic attributes at the individual, species, genus, and family levels with structural metrics provides a more comprehensive framework for understanding the ecological organization of tropical montane forests [8,9,10].

In Peru, much of the available information on the floristic composition and structure of montane forests comes from the central and southern regions of the country, whereas studies from the Department of Amazonas remain comparatively limited and geographically localized [7,8,26,27]. Although previous studies have documented tree diversity, floristic composition, and forest structure in several localities of Amazonas, detailed ecological baselines based on standardized permanent 1 ha plots and supported by vouchered botanical collections remain limited [8,28]. In particular, there is a need for permanent-plot inventories that integrate standardized measurements of tree diversity and forest structure with rigorous taxonomic documentation, conservation assessments, and the identification of previously undocumented regional records. Comparing these local inventories with comparable permanent plots from other regions of Peru also provides a broader national context for understanding the floristic composition of montane forests in Amazonas.

The main objective of this study was to characterize and compare tree diversity, floristic composition, forest structure, and conservation status among three permanent 1 ha montane forest plots in Amazonas, northern Peru. A second objective was to place their floristic composition within a broader Peruvian context through comparison with other permanent montane forest plots established using comparable sampling protocols. Given that each sampled elevation was represented by a single permanent plot, the study was designed to explore ecological variation among plots spanning the sampled elevational range rather than to formally test the effect of elevation or the shape of an elevational response. Conservation status was additionally assessed to document species of conservation concern, endemic taxa, and previously undocumented regional records within the three study plots. Accordingly, we considered the following expectations exploratory: (i) tree diversity would differ among the three plots; (ii) floristic composition and the identity of dominant species and families would differ among plots; and (iii) forest structure would vary among plots, as reflected by differences in stem density, basal area, and diameter-class distributions.

## 2. Materials and Methods

### 2.1. Study Area

The study was conducted in three 1 ha permanent forest plots located in the montane forests of the Department of Amazonas, northern Peru, between 2169 and 2519 m a.s.l. (Figure 1). The plots evaluated were P-SI1 (2519 m a.s.l.; 6.116722° S, 78.209444° W; UTM 17M 808867 E, 9323092 N), P-SI2 (2169 m a.s.l.; 6.111111° S, 78.198889° W; UTM 17M 810050 E, 9323691 N), and P-OC1 (2347 m a.s.l.; 6.252222° S, 77.554444° W; UTM 18M 217346 E, 9308222 N).

To characterize the environmental conditions of the study area, mean annual temperature (BIO1) and annual precipitation (BIO12) were extracted for each permanent plot from the WorldClim version 2.1 database (1970–2000) at a spatial resolution of 30 arc-seconds (~1 km) [29]. Soil properties and the dominant World Reference Base (WRB) soil group were obtained from the SoilGrids 2.0 database [30]. These data are spatially modeled estimates with inherent predictive uncertainty and do not represent direct field measurements; therefore, they were used only to provide a general environmental characterization of the study sites and not as explanatory variables in the statistical analyses. Mean annual temperature ranged from 14.7 to 16.7 °C, whereas annual precipitation varied between 855 and 1050 mm among plots. According to SoilGrids 2.0, the dominant predicted WRB soil group across the three plots was Andosols, and the soils were characterized by moderately acidic conditions (pH 5.4–5.5), high soil organic carbon contents (69.4–94.8 g kg^−1^), moderate-to-high cation exchange capacity (25.0–29.0 cmolc kg^−1^), and loam to clay loam surface textures (Table 1).

Local topographic conditions at each plot were characterized using a digital elevation model (DEM) and the terrain function in the R package terra. The DEM had a spatial resolution of approximately 0.000686° (~76 m at the study latitude). Slope and aspect were derived using an eight-neighbor algorithm and expressed in degrees. Because the DEM resolution was similar to the dimensions of the 1 ha plots, these values were interpreted as local topographic estimates at the plot coordinates rather than plot-wide averages. Accordingly, the derived slope and aspect values may not be representative of topographic conditions across the entire 1 ha plot. P-SI1 occurs on sloping montane terrain and has a DEM-derived local slope of 10.4° with a northeastern aspect. P-SI2 had the steepest local slope among the three sites (20.7°, southeastern aspect), whereas P-OC1 had a local slope of 9.3° with a southwestern aspect.

The three permanent plots are located within communal lands owned and administered by local peasant communities. Current conservation is based on customary community agreements that prohibit commercial timber harvesting while allowing traditional low-impact uses, such as firewood collection, access through established trails, and occasional livestock grazing. Community members also conduct local surveillance to reduce illegal logging and maintain native forest cover. Commercial timber extraction is currently not permitted within the studied forests.

Plot locations were selected purposively rather than randomly or systematically. Sites were identified during field reconnaissance based on the presence of a sufficiently continuous area of montane forest to establish a permanent 1 ha plot, accessibility for repeated censuses, authorization and logistical support from local communities, and the feasibility of protecting and monitoring the plots over the long term. The three plots represent selected permanent forest sites within the sampled elevational range rather than a probability sample of the montane forests of northern Peru. The potential bias associated with site selection should be considered when interpreting the representativeness of the results.

The three plots differed in disturbance history and local forest characteristics. P-SI2 (2169 m a.s.l.) is located in a montane forest showing evidence of historical selective logging. According to information provided by local residents, timber extraction occurred approximately 70 years ago. During plot establishment, this historical disturbance was supported by field observations, including old cut stumps and signs of previous logging activities. No historical forest management records were available to quantify the intensity of the extraction, including the number or basal area of trees removed. No comparable evidence of historical commercial timber extraction was observed in P-SI1 or P-OC1. In contrast, P-OC1 is located in a relatively well-conserved montane forest characterized by the dominance of palms of the genus *Ceroxylon* in the upper canopy and by the marked floristic dominance of *Ceroxylon* aff. *quindiuense*. Canopy cover and openness were not quantitatively measured during plot establishment and therefore were not included as explanatory variables in the analyses.

The three permanent plots represent the sample montane forests within the 2169–2519 m a.s.l. elevational range sampled in the study region. Rather than sampling the entire Andean elevational gradient, the study focused on the sampled section of montane forest to evaluate changes in tree diversity, floristic composition, forest structure, and conservation status using standardized 1 ha permanent plots. Restricting the study to a relatively narrow elevational range also reduced variation associated with major climatic zones and vegetation belts, allowing differences among plots to be interpreted within a more comparable environmental context. Likewise, the use of standardized permanent plots facilitates direct comparisons with other long-term monitoring networks across the tropical Andes.

### 2.2. Sampling Design and Data Collection

Three 1 ha plots were established along the evaluated elevational gradient. Each permanent plot covered an area of 1 ha (100 × 100 m) and was subdivided into 25 contiguous 20 × 20 m subplots. Each 1 ha permanent plot was treated as an independent sampling unit for local among-plot comparisons as an independent sampling unit for local am*(n* = 3). The 25 subplots within each plot were spatially nested sampling units used for field organization and for describing within-plot structural variability; they were not treated as independent replicates of elevation or plot-level environmental conditions. Plot establishment, measurements, and data collection followed the protocols of the RAINFOR Forest Inventory Network [31]. For analyses of tree diversity, floristic composition, and forest structure, each 1 ha plot was considered a sampling unit.

Within each plot, all trees with a diameter at breast height (DBH) ≥ 10 cm were inventoried. This threshold was selected because it is the inclusion criterion commonly used in permanent tropical forest plot networks and facilitates standardized comparisons of tree diversity, floristic composition, stem density, basal area, biomass, and forest dynamics among sites [32,33,34]. For each individual, the subplot, family, species, DBH, and total height were recorded. Tree diameter at breast height (DBH) was measured at 1.3 m above ground using a diameter tape (Richter 283D), and tree height was measured using a Nikon Forestry Pro laser hypsometer, following the RAINFOR field protocol. For trees with buttresses or other stem irregularities at breast height, the measurement point was moved upward to a position above the irregularity where the stem had a regular form. For leaning trees, DBH was measured at 1.3 m along the stem from the rooting point. For forked trees, stems were treated according to the position of the fork relative to breast height: when the fork occurred above 1.3 m, the individual was measured as a single stem, whereas stems separating below breast height were measured separately. For multi-stemmed individuals, each qualifying stem was measured separately and associated with the same individual. Palms and tree ferns meeting the ≥10 cm diameter criterion were included in the census; stem diameter was measured at 1.3 m following the same standardized principle, avoiding local stem irregularities when present.

Total height was measured for all individuals with DBH ≥ 10 cm using a Nikon Forestry Pro hypsometer. A single measurement was routinely taken for each individual. When a measurement was considered unreliable because of poor visibility of the tree crown, steep terrain, obstacles, or an anomalous reading, the measurement was repeated from a different observation position until a reliable reading was obtained. This procedure was used as a field quality-control measure for height measurements [31].

Taxonomic identification was based on botanical specimens collected following standard dendrological methods. Specimens were deposited in the MOLF Forest Herbarium, and duplicates were deposited in the KUELAP Herbarium of the Universidad Nacional Toribio Rodríguez de Mendoza (UNTRM), Amazonas. Collector numbers and, where available, herbarium accession numbers for taxa reported as new regional records are provided in Table A1. Identifications were made using specialized taxonomic literature, dichotomous keys, comparisons with reference collections, and the support of Carlos Reynel Rodríguez, an expert with extensive experience and recognized expertise in the dendrology and taxonomy of Peruvian trees. Scientific names were subsequently verified and updated using Tropicos [35] and Plants of the World Online [36]. Whenever possible, fertile botanical material was collected to facilitate taxonomic identification and the preparation of voucher specimens. All collections were conducted under an official collecting permit issued by the Peruvian National Forest and Wildlife Service (SERFOR). Voucher specimens of threatened species, including *Podocarpus oleifolius*, *Cedrela montana*, *Persea brevispes*, and *Mollinedia racemosa*, consisted only of limited fertile material collected for taxonomic purposes and did not involve the removal of whole individuals or activities that could compromise natural populations.

### 2.3. Diversity, Floristic Composition, and Forest Structure Analyses

Before calculating species richness and constructing the species–abundance matrix, taxonomic names were standardized using Tropicos and Plants of the World Online (POWO). Synonyms were reconciled with their currently accepted names, and spelling variants and nomenclatural inconsistencies were standardized to avoid duplicate taxonomic entities. No infraspecific taxa were present in the final analytical dataset. Morphospecies were assigned unique identifiers (e.g., *Genus* sp. 1 and *Genus* sp. 2) and treated as distinct taxonomic units in richness and abundance calculations. Tentative identifications (e.g., cf. and aff.) were likewise retained as distinct taxonomic units when they represented distinguishable entities in the botanical collections. The same standardized taxonomic dataset was used to calculate species richness and construct the abundance matrix for floristic analyses.

Alpha diversity in the study plots was evaluated using Hill numbers of orders q = 0, q = 1, and q = 2, corresponding to species richness, the exponential of Shannon entropy, and the inverse Simpson concentration, respectively [37,38]. The parameter q determines the sensitivity of the diversity measure to species relative abundances: q = 0 gives equal weight to all species, q = 1 accounts for their relative abundances, and q = 2 gives greater weight to dominant species [39]. Sample-size-based curves were extrapolated to a common endpoint of 1200 individuals. This endpoint was selected to allow comparison among the three assemblages at a common sample size while avoiding excessive extrapolation beyond the observed sample sizes and keeping extrapolation below twice the observed sample size of the smallest assemblage [39]. Sampling completeness was evaluated using sample coverage [39,40], which exceeded 97% in all three plots. Sample coverage was high across all three plots (97.7–98.1%), indicating that the inventories captured a large proportion of the estimated assemblage completeness. Nevertheless, high sample coverage does not imply complete detection of all species, and rare species may remain underrepresented or undetected. The 95% confidence intervals were estimated using 200 bootstrap replicates [39]. Sample coverage was used to assess sampling completeness, and rarefaction and extrapolation curves were used to compare diversity among plots under a standardized sampling effort. The Shannon–Wiener, Simpson, and Fisher’s alpha diversity indices were also calculated to facilitate comparisons with previous studies conducted in Peruvian montane forests.

Forest structure was described using stem density (individuals ha^−1^), basal area (m^2^ ha^−1^), and diameter and height distributions. Individuals were grouped into 10 cm diameter classes and 5 m height classes, following the same criterion used in 1 ha permanent plots established in Peruvian montane forests [27], thereby allowing comparisons of forest structure among different sites. Diameter inequality among trees was quantified using the Gini coefficient (G), calculated from the individual DBH values of all trees in each 1 ha plot. The Gini coefficient ranges from 0 (all trees have identical diameters) to 1 (maximum diameter inequality), providing a quantitative measure of inequality in tree diameters. Higher values indicate greater diameter inequality; however, their ecological interpretation is context-dependent and should not be considered a direct measure of overall forest structural complexity or stability. Confidence intervals (95%) were estimated using non-parametric bootstrap resampling with 2000 iterations. Differences among plots were evaluated by comparing bootstrap confidence intervals.

To identify the species and families with the greatest ecological importance, the Importance Value Index (IVI = RD + RF + RDo) was used, where RD represents relative density, RF relative frequency, and RDo relative dominance [41]. Relative density was calculated as the number of individuals of a species divided by the total number of individuals in the plot × 100, whereas relative dominance was calculated as the total basal area of a species divided by the total basal area of all species in the plot × 100. The absolute frequency of each species was determined within each 1-ha plot from the number of 20 × 20 m subplots in which it occurred, out of 25, and relative frequency was calculated as the frequency of the species divided by the sum of the frequencies of all species within each 1-ha plot × 100. For families, the Family Importance Value Index (FIVI = RD + RF + RDo) was used, calculated using the same components at the family level [42]. Because each relative component ranges from 0 to 100, both IVI and FIVI have a theoretical range of 0 to 300; the indices were neither divided by three nor rescaled as percentages.

Floristic composition was evaluated based on the relative abundance of families, genera, and species. Information on conservation status and geographic distribution was evaluated separately according to the purpose of each source. National conservation status was verified using Supreme Decree No. 043-2006-AG [21], whereas endemism was assessed using the 2006 Red Book of Endemic Plants of Peru [17] and the Catalogue of Trees of Peru [19]. Global extinction risk was evaluated independently using the IUCN Red List (2026) [43], considering the categories Critically Endangered (CR), Endangered (EN), Vulnerable (VU), Near Threatened (NT), and Least Concern (LC). CITES listing status was verified using the corresponding list [44]. These systems serve different purposes and were therefore interpreted independently. The Peruvian national classification reflects the legal conservation status of species under national legislation, the IUCN Red List provides a standardized assessment of global extinction risk, and CITES regulates international trade in listed species rather than directly classifying extinction risk. Consequently, a species may have different categories or status across these systems, and no attempt was made to reconcile them into a single conservation category. Species occurrence records and geographic distributions were cross-checked against specialized floristic literature and taxonomic sources, including Brako and Zarucchi [22], Vásquez et al. [19], and Tropicos [35], and subsequently verifies using the GBIF and speciesLink databases [45,46]. The latter two were used exclusively to verify occurrence records and geographic distributions and not to assign conservation categories. Information from the different assessment sources was not combined into a single conservation classification. A species was considered a new record for the Department of Amazonas when its occurrence in Amazonas had not been previously documented in the sources consulted, specifically Brako and Zarucchi [22], Vásquez et al. [19], Tropicos [35], GBIF [45], and speciesLink [46].

To provide a regional context for the floristic analyses, we selected reference plots representing Peruvian montane forests that were established using comparable sampling protocols. Specifically, the selected plots correspond to permanent 1 ha forest plots (or equivalent standardized inventories) with a minimum inclusion criterion of DBH ≥ 10 cm and available floristic data identified to the species level. The plots span a broad elevational range and represent the principal montane forest regions currently documented in Peru, allowing comparisons between the three Amazonas plots and other representative Andean forests using a standardized methodological framework (Table A2). For the nine reference plots, individual-level tree records (stem-level data) were available, including taxonomic identification and DBH measurements. Thus, abundance values used in the regional floristic comparison were not extracted from published diversity indices, figures, or summary statistics. The individual records from the nine reference plots and the three plots established in this study were standardized at the genus level and aggregated by plot to construct a plot × genus abundance matrix for the NMDS analysis. Floristic composition among plots was compared using non-metric multidimensional scaling (NMDS) based on Bray–Curtis dissimilarities calculated directly from a plot × genus matrix of raw, untransformed abundances. Each matrix cell represented the number of individuals of a given genus recorded in each plot, with absences assigned a value of zero. Abundances were not converted to relative abundances, and no square-root, logarithmic, or other transformation or standardization was applied before calculating Bray–Curtis dissimilarities; automatic transformation in metaMDS was also disabled (autotransform = FALSE). Rare genera were retained without applying a minimum abundance or occurrence threshold; only genera with zero total abundance across all plots were excluded. The NMDS was performed in two dimensions (k = 2) using the metaMDS function in the vegan package with the default convergence criteria of the monoMDS engine. A maximum of 500 random starts was allowed (trymax = 500), and a fixed random seed (set.seed(123)) was used to ensure reproducibility. A total of 20 tries were performed, and the best solution was repeated eight times. Genus scores were calculated as weighted averages (wascores = TRUE). The final two-dimensional solution had a stress value of 0.068. This stress value (<0.1) indicates a good representation of the dissimilarity relationships among plots [47]. Elevation and species richness were fitted to the ordination using the envfit function in the vegan package [48], and the significance of each association was assessed using 999 permutations (*p* < 0.05). All statistical analyses were performed in R version 4.4.3 [49] using the iNEXT and vegan packages.

Because the three 1 ha permanent plots constituted the only independent sampling units for the local analysis (*n* = 3), inferential statistical tests were not used to compare forest structural attributes among plots. The 25 subplots nested within each plot were not treated as independent replicates, as doing so would constitute pseudoreplication [50]. Structural variables were therefore summarized descriptively. Species diversity was evaluated using Hill numbers (q = 0, 1, and 2) and 95% confidence intervals generated by iNEXT. These confidence intervals quantify sampling uncertainty associated with individuals within each observed assemblage and were used only to describe and compare diversity among the three plots; they do not provide independent replication of elevation and were not interpreted as a statistical test of an elevation effect. The broader floristic comparison with independent reference plots from Peru (Table A2) was evaluated separately using multivariate analyses. Statistical significance for permutation-based analyses was assessed at α = 0.05.

## 3. Results

### 3.1. Alpha Diversity Among Plots

A total of 147 species were recorded across the three plots combined. At the plot level, observed species richness (Hill number, q = 0) ranged from 49 to 67 species (Table 2). P-OC1 showed the highest taxonomic richness, with 67 species, 48 genera, and 33 families, whereas P-SI2 had the lowest species richness, with 49 species, 36 genera, and 27 families. P-SI1 had 51 species, 30 genera, and 22 families.

Sample-size-based rarefaction and extrapolation curves based on Hill numbers showed variation in diversity among plots (Figure 2a). For q = 0, corresponding to species richness, P-OC1 had the highest observed richness (67 species), followed by P-SI1 (51) and P-SI2 (49). The same ranking was maintained for q = 1, with values of 24.6, 20.8, and 17.2, respectively, and for q = 2, with values of 13.6, 12.1, and 10.7. After extrapolating the curves to a common endpoint of 1200 individuals, this ranking was maintained, with estimated values of 71.6, 62.0, and 57.8 for q = 0; 24.9, 21.6, and 17.5 for q = 1; and 13.7, 12.2, and 10.7 for q = 2 in P-OC1, P-SI1, and P-SI2, respectively.

Coverage-based curves maintained the same ranking among plots (Figure 2b). Sample coverage was high and similar among the three plots, reaching 98.07% in P-OC1, 97.72% in P-SI1, and 97.73% in P-SI2, indicating similar levels of sample completeness among the three observed assemblages. At these coverage levels, differences were pronounced for q = 0 and decreased toward q = 2, where the curves converged.

### 3.2. Floristic Composition and Species of Conservation Interest

The tree communities recorded in the study plots comprised a total of 147 species, 75 genera, and 46 families (Table A3). Of the 2336 individuals recorded, 558 (23.9%) had some degree of taxonomic uncertainty, including 342 individuals (14.6%) identified only to genus level (sp.), 204 (8.7%) assigned by taxonomic affinity (aff.), and 12 (0.5%) requiring confirmation (cf.). Floristic composition differed among the three plots in both abundance and taxonomic richness of families and species (Figure 3). Lauraceae was the most species-rich family, with 25 species and morphospecies recorded across the three plots combined, and was also among the most abundant. Cyatheaceae and Euphorbiaceae reached their highest abundances in P-SI2, whereas Arecaceae predominated in P-OC1, and Clusiaceae and Cunoniaceae predominated in P-SI1 (Figure 3a,c).

At the species level, differences were also observed among plots. *Aiouea montana* (Sw.) R. Rohde was one of the most abundant species in P-SI2 and P-OC1. In P-SI1, *Weinmannia pinnata* L. and *Alchornea grandiflora* Müll.Arg. were among the most abundant species. In contrast, *Ceroxylon* aff. *quindiuense* was highly abundant in P-OC1, where it represented a characteristic component of the forest evaluated (Figure 3b).

Several taxa of conservation concern were recorded across the three plots (Table 3). According to the Peruvian national classification of threatened wild flora species [21], *Podocarpus oleifolius* was categorized as Critically Endangered (CR), *Persea brevipes* as Endangered (EN), and *Cedrela montana* as Vulnerable (VU). In addition, the tree ferns *Cyathea delgadii* and *Dicksonia sellowiana* are listed in CITES Appendix II [44]. According to the IUCN Red List (2026), most of the assessed taxa were classified as Least Concern (LC), representing 71.8% of the taxa evaluated. In addition, ten species represented new regional records for the Department of Amazonas, Peru. Accepted names, voucher specimen information, collection locality and plot, the authority responsible for identification, previously documented distribution, and evidence supporting their status as first records for Amazonas are presented in Table A1.

### 3.3. Forest Structure and Dominance of Species and Families

The horizontal and vertical structures of the tree communities varied across the sampled elevational gradient (Table 4).

Across the three plots, diameter distributions followed an inverted J-shaped pattern, characterized by a high concentration of individuals in the smaller diameter classes and a progressive decline toward larger diameter classes (Figure 4a). Height distributions were generally unimodal across the three plots (Figure 4b). The modal height class was 5–10 m in P-SI1 (314 individuals), whereas 10–15 m was the dominant class in both P-SI2 (358 individuals) and P-OC1 (322 individuals). Trees taller than 20 m were uncommon in all plots and were recorded mainly in P-OC1, indicating a greater contribution of taller canopy trees in this forest. Among the three plots, P-OC1 had the highest basal area (47.98 m^2^ ha^−1^). The diameter distributions of the three plots exhibited the typical inverted J-shaped pattern of tropical forests (Figure 4). This pattern was supported by the Gini coefficient, which revealed differences in diameter inequality among plots. P-SI1 showed the lowest structural inequality (G = 0.180; 95% CI: 0.167–0.191), whereas P-SI2 (G = 0.265; 95% CI: 0.234–0.296) and P-OC1 (G = 0.279; 95% CI: 0.268–0.289) exhibited greater diameter heterogeneity. Bootstrap comparisons indicated that P-SI1 differed significantly from both P-SI2 and P-OC1, whereas no significant difference was detected between P-SI2 and P-OC1.

Species ecological importance differed among plots according to the Importance Value Index (IVI). In P-SI2, *Myrcia megapaniculata* (IVI = 43.2) and *Aiouea montana* (IVI = 40.0) were the most ecologically important species. The relatively low maximum IVI in P-SI2 indicates that ecological importance was not strongly concentrated in a single species, but instead distributed among several taxa. In P-OC1, *Ceroxylon* aff. *quindiuense* had the highest IVI (43.8), followed by *Aiouea montana* (39.3). In contrast, P-SI1 was dominated by *Alchornea grandiflora* (IVI = 50.1) and *Weinmannia pinnata* (IVI = 36.0).

At the family level, the Family Importance Value Index (FIVI) also varied among plots (Figure 5b). Lauraceae had the highest values in P-SI2 (62.7) and P-OC1 (52.1). In P-OC1, Arecaceae also had a high FIVI (45.7). In P-SI1, the most ecologically important families were Clusiaceae (55.5), Euphorbiaceae (52.6), and Cunoniaceae (41.9).

### 3.4. Floristic Differentiation Among Plots

The NMDS analysis based on Bray–Curtis dissimilarity showed variation in the positions of the 12 plots in ordination space (Figure 6). P-SI1 and P-SI2 occupied relatively close positions, whereas P-OC1 was located farther from both, primarily along the second NMDS axis. Among the reference plots, P-SC and P-CCH were also located farther from the main group of plots in ordination space (Figure 6). These patterns were interpreted descriptively and not as evidence of statistically significant differences in floristic composition among plots.

The stress value was 0.068, corresponding to a low stress level, indicating that the ordination adequately represented the dissimilarity relationships among plots.

The vector-fitting analysis (envfit) showed a stronger association of elevation with ordination than species richness (R^2^ = 0.280 and R^2^ = 0.044, respectively). However, neither variable was significantly associated with the ordination based on 999 permutations (elevation: *p* = 0.223; species richness: *p* = 0.826). Elevation showed a stronger, but nonsignificant, association with the observed ordination pattern.

## 4. Discussion

### 4.1. Variation in Alpha Diversity Among Plots

Among the three plots, P-OC1 showed the highest tree diversity. However, because each elevation was represented by a single plot, this difference should be interpreted as a plot-level pattern rather than as evidence of an intermediate-elevation diversity maximum or a general elevational response. Similar non-monotonic patterns have been reported along some tropical montane forest gradients, whereas other studies have documented declines in woody plant diversity with increasing elevation [3,9,51,52]. These contrasting patterns indicate that relationships between tree diversity and elevation may vary among regions and depend on the environmental characteristics of individual sites.

The high species richness observed in P-OC1 coincided with a substantial representation of species associated with humid montane forests and the presence of *Ceroxylon* aff. *quindiuense*, a palm characteristic of Andean cloud forests [53]. In addition to elevation, several studies have indicated that local factors, such as microclimate, topography, and soil properties, can influence resource availability and, consequently, diversity patterns in tropical montane forests [9,54]. Although these variables were not evaluated in this study, the combination of these factors could help explain the higher diversity observed in P-OC1. The variability observed among plots suggests that tree diversity depends not only on elevation but also on the environmental conditions specific to each site.

### 4.2. Floristic Composition and Ecological Dominance

The floristic differences among the three plots suggest substantial taxonomic turnover at both family and species levels. Such compositional variation is characteristic of Neotropical montane forests, where changes in environmental conditions over relatively short spatial distances can be associated with marked shifts in tree-community composition [1,9,10]. The recurrent importance of Lauraceae is consistent with studies from montane forests of northern Peru, where this family constitutes a major component of the tree flora [7,8].

Differences in ecological dominance among plots further suggest that floristic turnover involved changes not only in taxonomic composition but also in the relative structural importance of taxa. In this context, the prominence of *Ceroxylon* aff. *quindiuense* in P-OC1 is ecologically relevant because species of *Ceroxylon* can form dense populations, attain considerable heights, and contribute substantially to the vertical structure of Andean montane forests [53,55]. *Ceroxylon* has also been regarded as a keystone taxon in Andean cloud forests because its species can form dense populations and produce abundant fleshy fruits consumed by a wide range of birds and mammals [53]. Recent evidence further highlights the importance of *Ceroxylon*-dominated forests for diverse faunal communities [56]. The presence of *Ceroxylon* may therefore contribute to the structural and ecological characteristics observed in P-OC1, although these relationships were not formally tested in the present study.

The recurrent ecological importance of families such as Lauraceae, Myrtaceae, and Cunoniaceae despite species turnover may reflect the persistence of family-level ecological strategies across heterogeneous montane environments. Environmental filtering can favor taxa with functional attributes suited to the increasingly harsh conditions of tropical montane forests, whereas tropical niche conservatism may constrain the distribution of lineages according to their inherited environmental tolerances. Studies across Andean montane forests have provided evidence consistent with these processes, including stronger environmental filtering toward higher elevations and the persistence of tropical lineages associated with phylogenetically conserved ecological strategies [3,15]. These mechanisms could contribute to the recurrent ecological importance of Lauraceae despite turnover among its constituent species. Indeed, previous studies have shown that Lauraceae can maintain high richness and abundance along elevational gradients despite marked changes in species composition [57]. However, because functional traits and phylogenetic structure were not evaluated in the present study, environmental filtering and niche conservatism should be regarded as plausible hypotheses rather than mechanisms directly demonstrated by our data.

Overall, the results suggest that floristic composition and patterns of ecological dominance in the montane forests evaluated are shaped by the interaction between elevational variation and environmental heterogeneity among sites. The persistence of certain taxonomic groups, together with the replacement of dominant species, highlights the high floristic heterogeneity characteristic of tropical montane forests.

### 4.3. Forest Structure

The inverted J-shaped diameter distributions observed in the three plots characterize stands with a predominance of individuals in smaller diameter classes. Although this pattern is commonly reported in tropical forests [58,59], it should not be interpreted as evidence of continuous regeneration, stable population replacement, or demographic stability, because such inferences require repeated censuses and direct estimates of recruitment and mortality. Differences in diameter inequality among plots further indicate variation in stand size structure; however, the ecological meaning of the Gini coefficient is context-dependent and should not be interpreted independently as evidence of forest maturity, complexity, or stability.

The vertical structure observed in the plots is broadly consistent with that reported for montane forests of the northeastern Peruvian Andes [28]. In particular, palms of the genus *Ceroxylon* can contribute substantially to forest vertical structure because of their capacity to attain considerable heights and form conspicuous components of Andean montane canopies [53,55].

The structural differences among plots may reflect the influence of multiple site-specific factors in addition to elevation. Historical disturbance and local topographic conditions are particularly relevant because selective logging can produce persistent changes in stand structure and species composition [60], while topographic variation can influence drainage, soil properties, hydrology, and resource availability [16]. Historical selective logging may also have contributed to the diversity and floristic composition currently observed in P-SI2. Canopy opening and associated changes in resource availability following logging can promote the establishment of light-demanding or early-successional species, potentially allowing species richness to recover while altering species evenness, relative abundances, and community composition [61,62]. Recovery of species richness does not necessarily imply recovery of the original floristic composition, as compositional changes may persist for several decades after logging [62,63]. Therefore, the present diversity and composition of P-SI2 may partly reflect a long-term legacy of historical disturbance. However, because pre-logging data, logging intensity, and a comparable unlogged control are unavailable, the magnitude and direction of this potential effect cannot be determined from the present study. Likewise, because detailed topographic conditions were not quantified, their relative contribution to the observed structural variation cannot be determined.

More broadly, the current observational design does not allow the independent effects of elevation, topography, and disturbance history to be disentangled, because these factors were not independently replicated or experimentally controlled. Their individual contributions to the observed differences in diversity, floristic composition, and forest structure therefore remain unresolved. A replicated design, including multiple plots within each elevational band and spanning comparable topographic and disturbance conditions, would be required to evaluate their relative effects.

### 4.4. Floristic Differentiation and Spatial Patterns

The inclusion of reference plots established using comparable permanent-plot protocols allowed the floristic composition of the three Amazonas forests to be interpreted within a broader regional context. Because these plots span the main elevational and geographic sectors currently represented by permanent montane forest inventories in Peru, the NMDS provided a regional framework for evaluating floristic affinities among Peruvian montane forests. Although the ordination revealed differences in floristic composition among plots, neither elevation nor species richness showed a significant association with the ordination. Therefore, the observed floristic differentiation cannot be attributed to elevation alone and may also reflect environmental, historical, and biogeographic factors that were not explicitly evaluated in this study.

The differentiation observed among plots within similar elevational ranges further suggests that compositional variation is not determined by elevation alone. Comparable patterns have been reported in montane forests, where floristic composition varies among sites with different environmental conditions [12]. The interpretation of diversity patterns may also depend on the spatial scale at which they are evaluated [52].

Comparison with permanent plots from different regions of Peru provided a regional context for our results and showed that the floristic differentiation observed in Amazonas falls within the compositional variation reported for tropical montane forests. Given the limited number of plots evaluated, these patterns should be interpreted as representative of the sites analyzed rather than as regional inferences. The interpretation of floristic turnover should also consider the taxonomic resolution of the dataset. Although most individuals were identified to species level, 23.9% were recorded with some degree of taxonomic uncertainty (sp., aff., or cf.). These unresolved or provisional identifications may influence estimates of species-level turnover by obscuring differences among closely related taxa or introducing uncertainty in species assignments. Nevertheless, the regional NMDS analysis was conducted at the genus level, which reduces the influence of uncertain species-level identifications on the broader compositional patterns observed. The observed variation also supports the need for additional permanent plots and long-term monitoring to better understand the environmental factors associated with differences among montane forest communities.

### 4.5. Ecological and Conservation Implications

Montane forests of northern Peru are ecosystems of high ecological importance because of their floristic diversity, the presence of species of conservation concern, and remaining gaps in botanical knowledge across the region [17,19,64]. *Critoniopsis jelskii*, a species endemic to Peru [17,19], was recorded in the study plots. This species was not considered a new regional record for the Department of Amazonas. In contrast, ten species represented new regional records for Amazonas, as documented in Table A1.

The differences observed between Peru’s national threat categories and the IUCN Red List may reflect the different spatial scales and assessment approaches used by each system. The IUCN assesses extinction risk at the global scale, whereas national assessments consider the status of species within Peru, where local population status and threats may differ from those observed across the species’ global range [20,65]. These differences in scale may result in different threat categories for the same species [65,66]. In this study, *Podocarpus oleifolius* is classified as Critically Endangered (CR) under Peruvian legislation but as Least Concern (LC) on the IUCN Red List, whereas *Cyathea delgadii* is categorized as Vulnerable (VU) in Peru but has no assessment available on the IUCN Red List. These discrepancies highlight the importance of considering national and global conservation assessments together, as national assessments can provide complementary information on extinction risk and conservation priorities at the national level [65].

Our study is based on three permanent plots and therefore does not capture the full environmental heterogeneity of the northern Peruvian Andes. Consequently, the observed patterns should be interpreted as representative of the sampled forests rather than as general responses to elevation. However, the use of standardized 1 ha permanent plots, together with comparisons within a regional network of comparable inventories, provides a framework for evaluating floristic and structural variation in a broader Andean context. Future studies incorporating additional permanent plots across broader environmental gradients will improve our understanding of the factors associated with montane forest diversity and dynamics. The relatively narrow elevational range covered by the three study plots (2169–2519 m a.s.l.) limits the extent to which the observed patterns can be generalized to the entire elevational gradient of montane forests in northern Peru. Therefore, our results should be interpreted as a description of variation within the sampled montane forest belt rather than as evidence of a general response of tree diversity, floristic composition, or forest structure to elevation. This limitation is particularly important because local factors, such as topography, soil conditions, and disturbance history, may contribute to variation among forests occurring at similar elevations. The comparison with permanent plots distributed across a broader elevational range in Peru provides a regional context for the observed patterns but does not substitute for replicated sampling across the entire elevational gradient. A research design specifically aimed at evaluating the effects of elevation would require multiple independent permanent plots replicated across several elevational bands, allowing variation associated with elevation to be distinguished from site-specific variation. Such a design would allow the application of appropriate inferential methods, including analysis of variance or covariance when their assumptions are met, or hierarchical models that account for the nested spatial structure of the data. The sampling design of the present study does not provide this level of replication; therefore, we did not make causal inferences regarding the effect of elevation. Additional permanent plots distributed across broader and replicated elevational bands will be needed to formally evaluate the effects of elevation and distinguish them from local environmental and historical influences.

From a conservation perspective, the floristic and structural differences among the three plots indicate that conservation actions should not focus exclusively on the most species-rich site, but should maintain the complementarity represented by forests with different species composition, structural attributes, and conservation-relevant taxa. Particular attention should be given to palm-dominated forests such as P-OC1, given the ecological importance of *Ceroxylon* and the occurrence of species of conservation concern, while historically disturbed forests such as P-SI2 would benefit from preventing further logging and maintaining conditions that facilitate long-term recovery. Management priorities should therefore include maintaining forest cover and connectivity, preventing additional selective logging and forest conversion, protecting populations of threatened and endemic species, and continuing permanent-plot monitoring. Because the study plots occur on communal lands, these actions should be developed jointly with local communities by strengthening existing community agreements, participatory monitoring and surveillance, and the incorporation of local knowledge into conservation decisions.

Future research should increase the number of permanent plots and integrate repeated tree censuses with measurements of environmental variables. Repeated censuses would allow the estimation of recruitment, mortality, diameter growth, changes in biomass, and temporal variation in species and family dominance. Likewise, spatially explicit analyses of soil physicochemical properties—including pH, texture, organic carbon, nitrogen, phosphorus, exchangeable bases, cation exchange capacity, and aluminum—would allow an assessment of whether local edaphic heterogeneity is associated with differences in composition and structure among plots located at similar elevations. Continuous or high-frequency climatic measurements, particularly of air and soil temperature, precipitation, relative humidity, vapor pressure deficit, cloud immersion, and soil moisture, would contribute to a better understanding of the relationships between climatic variability and forest dynamics. Integrating these observations with canopy structure data obtained from repeated field measurements or LiDAR would provide a more comprehensive understanding of the environmental factors associated with diversity, biomass, and structural changes in the montane forests of northern Peru.

## 5. Conclusions

Our results document variation in tree diversity, floristic composition, and forest structure among the three permanent montane forest plots evaluated in northern Peru. Across the three plots, alpha diversity did not show a monotonic ordering across the sampled elevations, with P-OC1 (2347 m a.s.l.) showing the highest observed richness and diversity. Given that each elevation was represented by a single plot, this pattern should be interpreted as variation among the three sampled plots rather than as evidence of a general elevational trend. Rather, they describe ecological variation among the three sampled plots, suggesting that elevation alone may not account for the observed variation and that additional local factors may also be important. The regional comparison with permanent plots established using comparable methodologies further showed substantial floristic variation among the plots examined, including among sites occurring at similar elevations.

The regional comparison placed the three study plots within the broader context of Peruvian montane forests and highlighted the high floristic heterogeneity of these ecosystems. The occurrence of species of conservation concern and new regional records reinforces the biological importance of the montane forests of northern Peru. Continued monitoring of these permanent plots through repeated censuses will provide the temporal data needed to quantify recruitment, mortality, tree growth, and changes in forest structure and composition. Expanding the permanent-plot network across broader and replicated elevational bands, together with measurements of soil, topography, and climate, will be necessary to formally evaluate the effects of elevation and other environmental drivers on montane forest diversity and structure.

Expanding the network of permanent plots using standardized protocols, together with the incorporation of environmental variables such as soil physicochemical properties and microclimate, will provide a more precise understanding of the ecological processes that structure tropical montane forests in northern Peru. This information provides a baseline for biodiversity documentation and long-term monitoring of the three permanent forest plots.

## Figures and Tables

**Figure 1:**
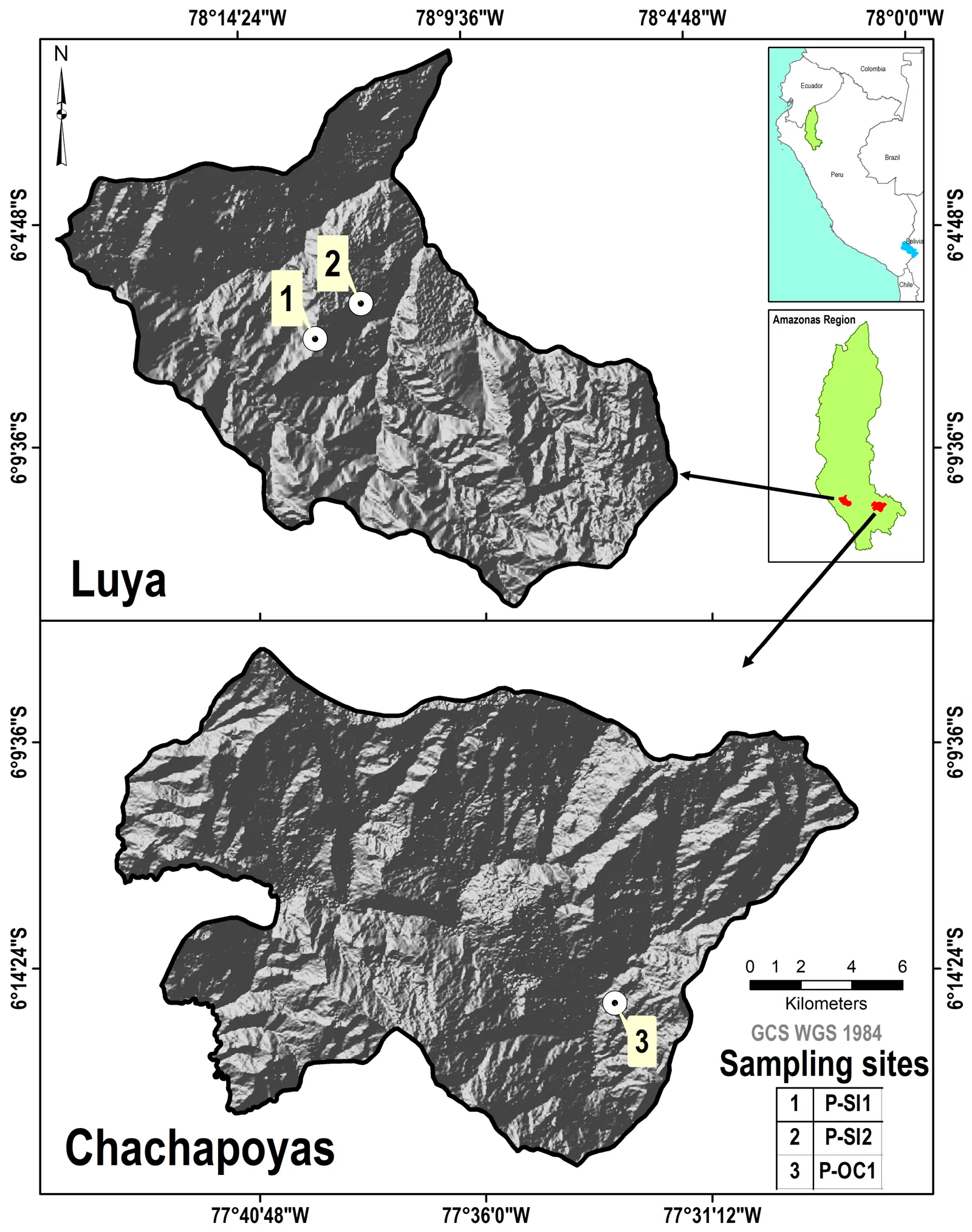
Location map of the study area and sampling sites in the provinces of Luya and Chachapoyas, Amazonas Region, northern Peru. The map illustrates terrain relief using hillshade visualization and the location of the permanent plots: P-SI1 and P-SI2 in Luya Province, and P-OC1 in Chachapoyas Province. Insets show the location of the Amazonas Region within Peru and the relative position of the study sites. The coordinate reference system used was GCS WGS 1984.

**Figure 2:**
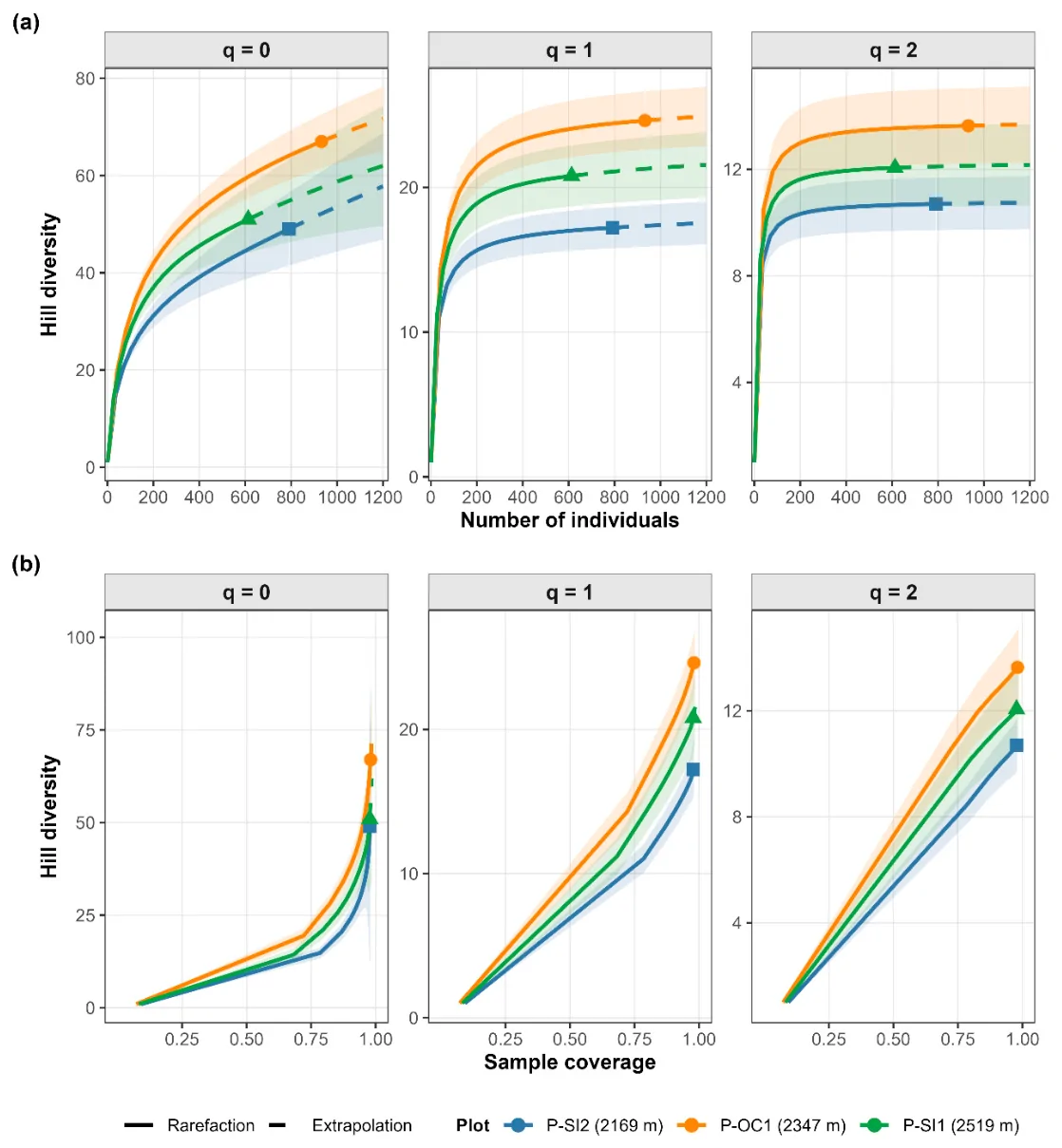
Rarefaction and extrapolation curves based on Hill numbers for the three permanent montane forest plots evaluated. (**a**) Sample-size-based rarefaction and extrapolation curves; (**b**) sample-coverage-based rarefaction and extrapolation curves. The panels show diversity orders q = 0, q = 1, and q = 2. Solid lines represent rarefaction, dashed lines represent extrapolation, and shaded areas indicate 95% confidence intervals.

**Figure 3:**
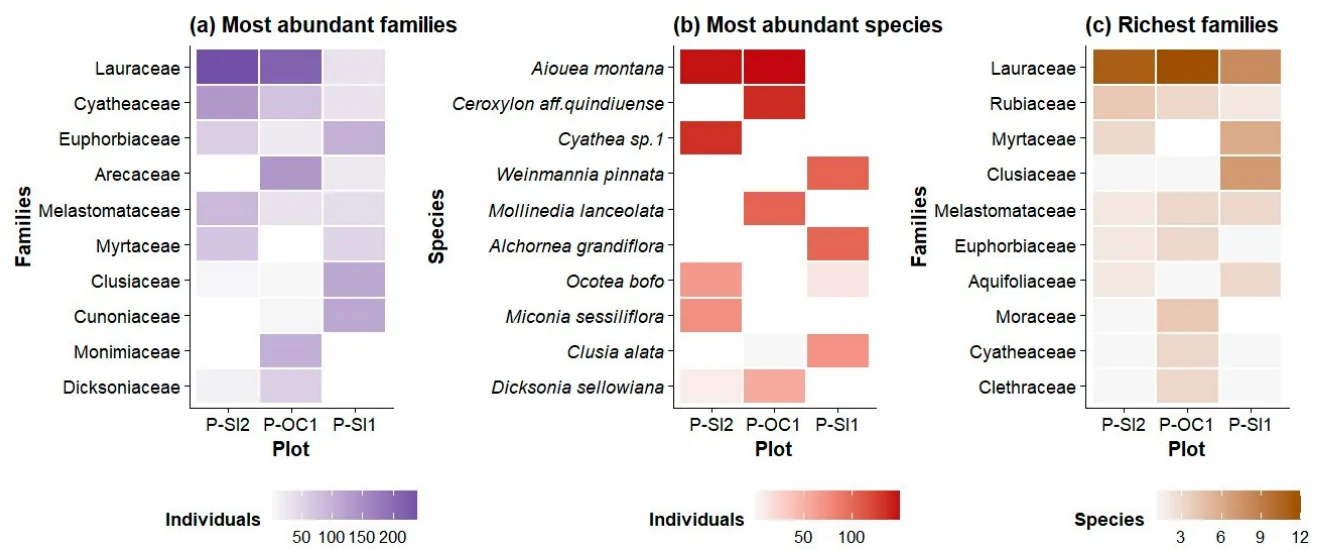
Floristic composition and taxonomic richness of permanent montane forest plots in northern Peru. (**a**) Most abundant families, (**b**) most abundant tree species, and (**c**) families with the highest species richness.

**Figure 4:**
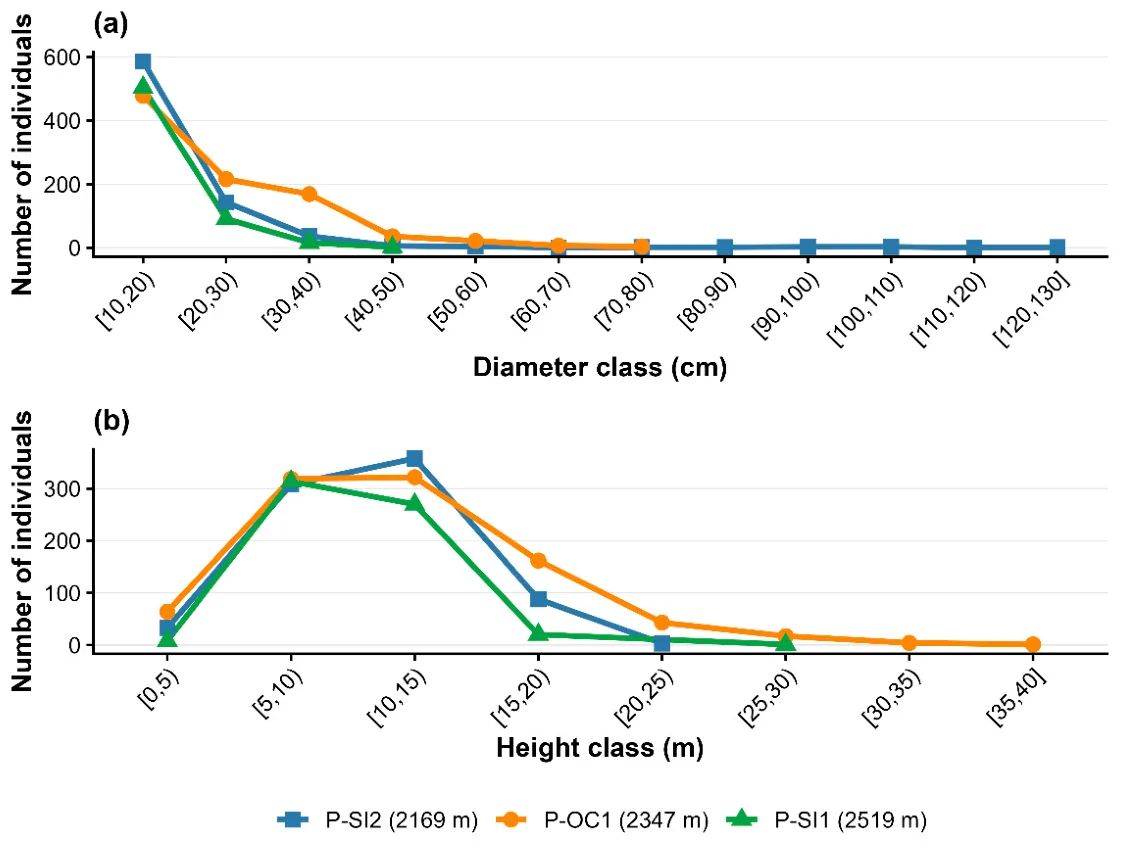
Distribution of individual trees in three 1 ha permanent plots established in montane forests of northern Peru. (**a**) Diameter-class distribution using 10 cm intervals; (**b**) height-class distribution using 5 m intervals. Height distributions were unimodal, with modal classes of 5–10 m in P-SI1 and 10–15 m in P-SI2 and P-OC1.

**Figure 5:**
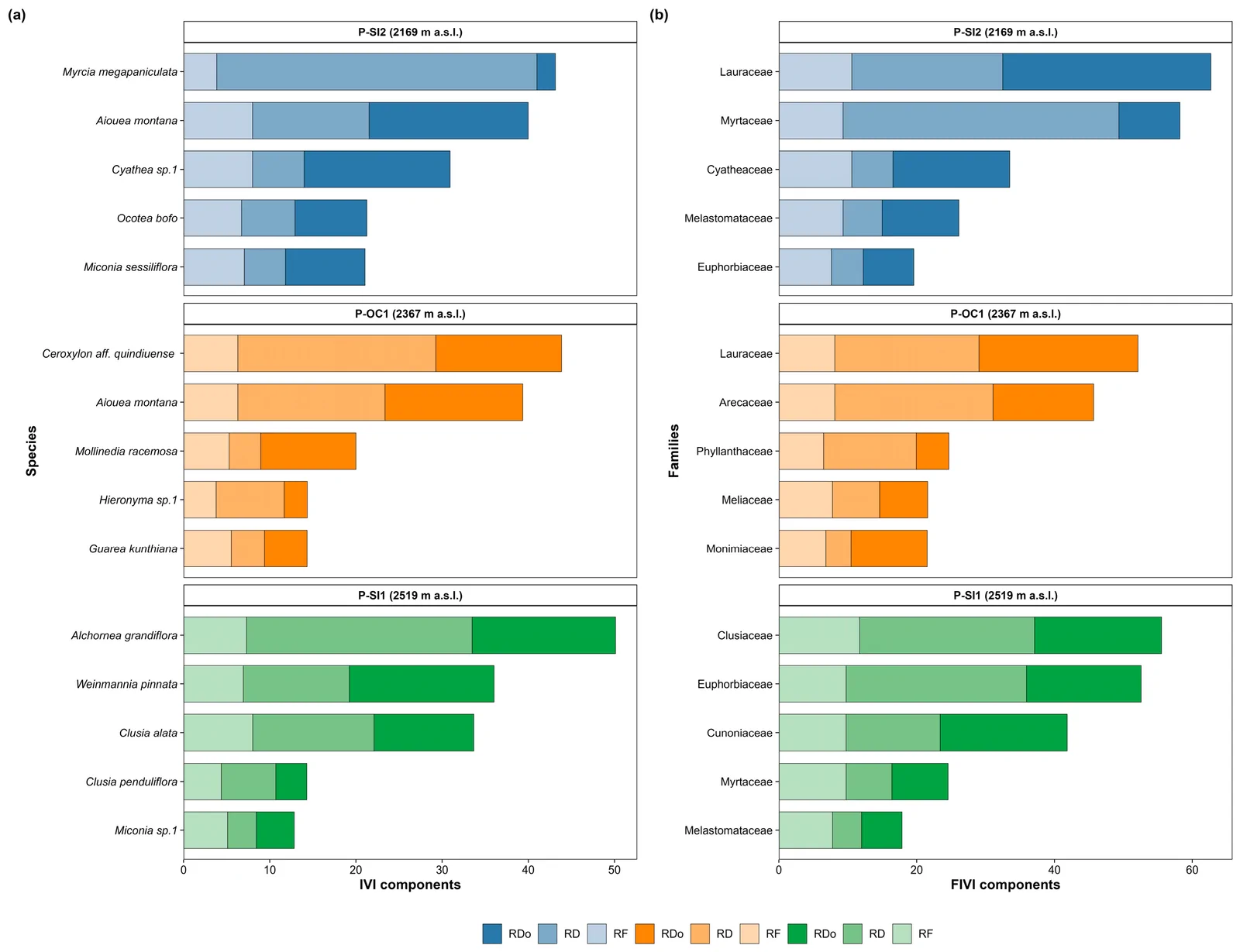
Importance Value Index (IVI) of the five highest-ranking species (**a**) and Family Importance Value Index (FIVI) of the five highest-ranking families (**b**) in each permanent plot. Horizontal stacked bars show the contributions of relative density (RD), relative frequency (RF), and relative dominance (RDo) to IVI and FIVI. RD represents the proportion of individuals belonging to each taxon, RF represents its frequency of occurrence across the 25 subplots relative to the summed frequencies of all taxa, and RDo represents the proportion of total basal area contributed by each taxon. Each component ranges from 0 to 100%, whereas IVI and FIVI range from 0 to 300. Only the five highest-ranking taxa in each plot are displayed for clarity.

**Figure 6:**
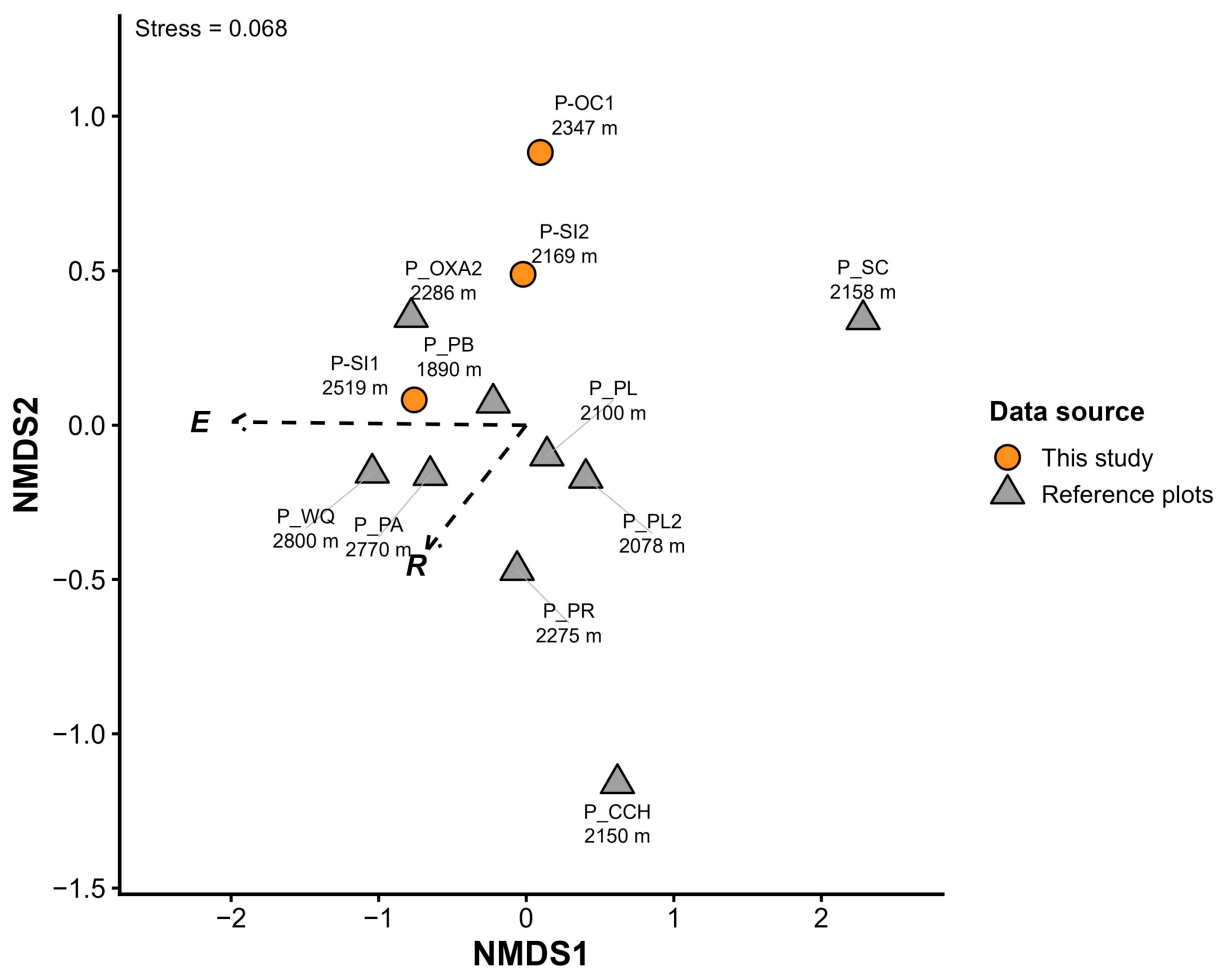
Non-metric multidimensional scaling (NMDS) ordination based on Bray–Curtis dissimilarity of genus-level abundance across 12 permanent montane forest plots in Peru. Circles represent the plots evaluated in this study, whereas triangles indicate the reference plots included for regional comparison. The arrows represent the fitted vectors for elevation (E) and species richness (R) obtained using the envfit function. Stress = 0.068.

**Table 1:** Geographic, climatic, topographic, and edaphic characteristics of the three permanent montane forest plots in northern Peru.

Plot	Elevation (m a.s.l.)	Slope (°)	Aspect (°; Direction)	MAT (°C)	MAP (mm yr^−1^)	WRB Soil Group	pH (H_2_O)	SOC (g kg^−1^)	CEC (cmolc kg^−1^)	Clay (%)	Sand (%)	Silt (%)	Texture
P-SI2	2169	20.7	126.1 (SE)	13.9	1092	Andosols	5.4	90.3	29	26	39.9	34.1	Loam
P-OC1	2347	9.3	236.4 (SW)	13.5	1097	Andosols	5.4	94.8	27.8	28.2	40.7	31	Loam
P-SI1	2519	10.4	31.8 (NE)	12.7	1063	Andosols	5.5	69.4	25	31.2	38.7	30.1	Clay loam

Note: MAT, mean annual temperature; MAP, mean annual precipitation; WRB, World Reference Base for Soil Resources; SOC, soil organic carbon; CEC, cation exchange capacity. Climatic variables were obtained from WorldClim v2.1 (1970–2000), and soil properties and WRB soil groups were obtained from SoilGrids. Slope and aspect are DEM-derived local estimates at the plot coordinates. Because the DEM spatial resolution (~76 m) is similar to the dimensions of the 1 ha plots (100 × 100 m), these topographic values represent local estimates rather than plot-wide means.

**Table 2:** Alpha diversity and taxonomic richness of the three permanent montane forest plots evaluated in northern Peru.

Variable	P-SI2	P-OC1	P-SI1
altitude (m a.s.l)	2169	2347	2519
Individuals	791	932	613
Families	27	33	22
Genera	36	48	30
Species	49	67	51
Species richness (Hill number, q = 0)	49	67	51
Hill number (q = 1)	17.2	24.6	20.8
Hill number (q = 2)	10.7	13.6	12.1
Shannon–Wiener index (H′)	2.85	3.20	3.03
Simpson Index (1 − D)	0.91	0.93	0.92
Fisher’s α	11.55	16.55	13.22

**Table 3:** Conservation status of tree and tree fern species of conservation concern recorded in the three permanent montane forest plots in northern Peru according to the IUCN Red List, Peruvian national legislation, and CITES.

Species	IUCN Red List [43]	National Legislation [21]	CITES-Peru [44]
*Podocarpus oleifolius D.* Don.	Least Concern (LC)	Critically Endangered (CR)	_
*Persea brevipes* Meins.	Endangered (EN)	_	_
*Cedrela montana* Moritz ex Turcz.	Vulnerable (VU)	Vulnerable (VU)	_
*Cyathea delgadii* Sternb.	_	Vulnerable (VU)	Appendix II
*Dicksonia sellowiana* Hook.	_	_	Appendix II
*Sciodaphyllum violaceum* (Cuatrec.) Lowry, G.M.Plunkett & M.M.Mora	Near Threatened (NT)	_	_
*Trichilia tomentosa* Kunth	Near Threatened (NT)	_	_
*Styloceras laurifolium* (Willd) Ex. Kunth	Least Concern (LC)	Endangered (EN)	_
*Ruagea glabra* Triana & Planch.	Least Concern (LC)	Endangered (EN)	_

**Table 4:** Structural attributes of the three permanent 1 ha plots evaluated in montane forests of northern Peru.

Variable	P-SI2	P-OC1	P-SI1
Mean DBH (cm)	18.6	22.69	15.61
Median DBH (cm)	15.1	19.35	13.8
Maximum DBH (cm)	192.3	78.5	47.1
Basal area (m^2^ ha^−1^)	33.97	47.98	13.23
Mean height (m)	10.46	11.52	9.65
Maximum height (m)	21	37	27
Modal height class	[10, 15)	[10, 15)	[5, 10)
Gini coefficient 95% CI (bootstrap)	0.265 0.234–0.296	0.279 0.268–0.289	0.18 0.167–0.191

## Data Availability

The data supporting the findings of this study are available from the corresponding author upon reasonable request.

## Appendix A

**Table A1:** Voucher information, collection localities, and previously documented distribution of tree species recorded as new departmental records for Amazonas, Peru.

Accepted Name	Family	Collector/Voucher No.	Herbarium Accession No.	Depository	Collection Locality	Plot	Determined by	Previously Known Distribution	Sources Consulted for Distribution
*Saurauia glabra* (Ruiz & Pav.) Soejarto	Actinidaceae	Rivera López/RR-540	KUELAP-6858	KUELAP	San Isidro, Conila, Luya	P-SI2	C. Reynel	CA, HU, LL, PA, UC	[19,22,35,45,46]
*Ilex danielis* Killip & Cuatrec.	Aquifoliaceae	Rivera López/RR-362	52510	MOLF	San Isidro, Conila, Luya	P-SI1	C. Reynel	CA, UC	[19,22,35,45,46]
*Sciodaphyllum weberbaueri* (Harms) Lowry, G.M.Plunkett & M.M.Mora	Araliaceae	Rivera López/RR-357	52507	MOLF	San Isidro, Conila, Luya	P-SI1	C. Reynel	HU, PA	[19,22,35,45,46]
*Clusia schultesii* Maguire	Clusiaceae	Rivera López/RR-560	KUELAP-6875	KUELAP	San Isidro, Conila, Luya	P-SI1	C. Reynel	CA, LL, PA	[19,22,35,45,46]
*Alchornea brittonii* Secco	Euphorbiaceae	Rivera López/RR-413	52530	MOLF	Ocol, Molinopampa, Chachapoyas	P-OC1	C. Reynel	JU, PA	[19,22,35,45,46]
*Nectandra oppositifolia* Nees & Mart. ex Nees	Lauraceae	Rivera López/RR-462	52544	MOLF	Ocol, Molinopampa, Chachapoyas	P-OC1	C. Reynel	LO, PA	[19,22,35,45,46]
*Ocotea argyrophylla* Ducke	Lauraceae	Rivera López/RR-372	52515	MOLF	San Isidro, Conila, Luya	P-SI1	C. Reynel	HU, JU, LO, SM, PA, UC	[19,22,35,45,46]
*Trichilia tomentosa* Kunth	Meliaceae	Rivera López/RR-456	52541	MOLF	Ocol, Molinopampa, Chachapoyas	P-OC1	C. Reynel	CA, LA, PI, TU	[19,22,35,45,46]
*Ficus gigantosyce* Dugand	Moraceae	Rivera López/RR-503	52554	MOLF	Ocol, Molinopampa, Chachapoyas	P-OC1	C. Reynel	CA, CU, PA, SM, UC	[19,22,35,45,46]
*Myrcia megapaniculata* A.R.Lourenço & Parra-Os.	Myrtaceae	Rivera López/RR-266	52449	MOLF	San Isidro, Conila, Luya	P-SI2	C. Reynel	CA, HU, MD, PA, LO	[19,22,35,45,46]

Note: Previously documented distribution refers to the departments of Peru in which each species was recorded in the sources consulted: Cajamarca (CA), Cusco (CU), Huánuco (HU), Junín (JU), Lambayeque (LA), La Libertad (LL), Loreto (LO), Madre de Dios (MD), Pasco (PA), Piura (PI), San Martín (SM), Tumbes (TU), and Ucayali (UC). Species distributions were checked against Brako and Zarucchi [22], Vásquez et al. [19], Tropicos [35], GBIF [45], and speciesLink [46]. Species whose occurrence in the Department of Amazonas had not been previously documented in the sources consulted were considered new departmental records. MOLF and KUELAP correspond to the herbaria where the voucher specimens were deposited.

**Table A2:** Richness, abundance and alpha diversity metrics of permanent montane forest plots used for regional comparisons in Peru.

Plot Codes	Department	Elevation (m a.s.l.)	Individuals (N)	Species (S)	Genus (G)	Families (F)	Shannon (H′)	Simpson (1 − D)	Fisher’s α
P-PB	AM	1890	640	152	60	39	4.26	0.97	23.74
P-PL2	JU	2078	680	155	87	45	NA	NA	39.68
P-PL	JU	2100	694	147	82	42	4.26	0.97	54.7
P-CCH	CA	2150	308	39	31	21	3.31	NA	11.83
P-SC	AM	2158	395	29	27	22	2.62	NA	7.21
P-SI2	AM	2169	791	49	36	27	2.85	0.91	11.25
P-PR	JU	2275	530	118	83	39	3.48	0.97	45.2
P-OXA2	PA	2286	584	72	46	34	3.36	0.94	NA
P-OC1	AM	2347	932	67	48	33	3.20	0.93	16.55
P-PSI1	AM	2519	613	51	30	22	3.03	0.92	13.22
P-PA	JU	2770	477	54	25	19	3.09	0.92	15.7
P-WQ	CU	2800	709	68	28	20	3.15	0.92	18.6

Note: Plot codes correspond to permanent plots located in five departments of Peru: Amazonas (AM), Cajamarca (CA), Pasco (PA), Junín (JU), and Cusco (CU). H′ = Shannon–Wiener diversity index; 1 − D = Simpson diversity index; Fisher’s α = Fisher alpha diversity index. NA indicates values not reported in the original sources. Data were compiled from published sources [7,8,27,67,68].

**Table A3:** List of tree species recorded in the three permanent montane forest plots of northern Peru.

Family	Genus	Species	P-OC1	P-SI1	P-SI2
Actinidaceae	Saurauia	*Saurauia glabra* Merr.	0	0	1
Aquifoliaceae	Ilex	*Ilex danielis* Killip & Cuatrec.	0	1	0
Aquifoliaceae	Ilex	*Ilex juttana* Loizeau & Spichiger	0	0	1
Aquifoliaceae	Ilex	*Ilex laureola* Triana & Planch.	0	0	1
Aquifoliaceae	Ilex	*Ilex laurina* Kunth	0	1	0
Aquifoliaceae	Ilex	*Ilex scopulorum* Kunth	1	0	0
Aquifoliaceae	Ilex	*Ilex* sp.1	0	1	0
Araliaceae	Oreopanax	*Oreopanax* aff. *pavonii* Seem.	1	0	0
Araliaceae	Sciodaphyllum	*Sciodaphyllum violaceum* (Cuatrec.) Lowry, G.M.Plunkett & M.M.Mora	0	0	1
Araliaceae	Sciodaphyllum	*Sciodaphyllum weberbaueri* (Harms) Lowry, G.M.Plunkett & M.M.Mora	0	1	0
Arecaceae	Ceroxylon	*Ceroxylon* aff. *quindiuense* (H. Karst.) H. Wendl.	1	0	0
Arecaceae	Ceroxylon	*Ceroxylon peruvianum* Galeano, Sanín & K.Mejia	0	1	0
Arecaceae	Ceroxylon	*Ceroxylon* sp.1	0	1	0
Asteraceae	Critoniopsis	*Critoniopsis jelskii* (Hieron.) H.Rob.	0	0	1
Asteraceae	Critoniopsis	*Critoniopsis* sp.1	1	0	0
Asteraceae	Vernonanthura	*Vernonanthura patens* (Kunth) H. Rob.	0	0	1
Buxaceae	Styloceras	*Styloceras laurifolium* (Willd) Kunth	1	0	0
Cardiopteridaceae	Citronella	*Citronella melliodora* (Sleumer) R.A.Howar	1	0	0
Chloranthaceae	Hedyosmum	*Hedyosmum spectabile* Todzia	0	0	1
Clethraceae	Clethra	*Clethra pedicellaris* Turcz.	0	0	1
Clethraceae	Clethra	*Clethra* aff. *peruviana* Szyszyl.	1	0	0
Clethraceae	Clethra	*Clethra revoluta* (Ruiz & Pav.) Spreng.	1	1	0
Clethraceae	Clethra	*Clethra* sp.1	1	0	0
Clusiaceae	Clusia	*Clusia alata* Planch. & Triana	1	1	0
Clusiaceae	Clusia	*Clusia penduliflora* Engl.	0	1	0
Clusiaceae	Clusia	*Clusia* sp.1	0	0	1
Clusiaceae	Clusia	*Clusia elliptica* Kunth	0	1	0
Clusiaceae	Clusia	*Clusia rosea* Jacq.	0	1	0
Clusiaceae	Clusia	*Clusia schultesii* Maguire	0	1	0
Clusiaceae	Clusia	*Clusia thurifera* Planch. & Triana	0	1	0
Clusiaceae	Tovomita	*Tovomita* sp.1	0	1	0
Cornaceae	Cornus	*Cornus peruviana* J. F. Macbr.	1	0	0
Cunoniaceae	Weinmannia	*Weinmannia ovata* Ruiz & Pav.	0	1	0
Cunoniaceae	Weinmannia	*Weinmannia pinnata* L.	0	1	0
Cunoniaceae	Weinmannia	*Weinmannia lentiscifolia* C. Presl	1	0	0
Cunoniaceae	Weinmannia	*Weinmannia* sp.1	0	1	0
Cyatheaceae	Cyathea	*Cyathea pungens* (Willd.) Domin	1	0	0
Cyatheaceae	Cyathea	*Cyathea delgadii* Sternb.	1	0	0
Cyatheaceae	Cyathea	*Cyathea* sp. 1	0	0	1
Cyatheaceae	Cyathea	*Cyathea* cf. *plicata* Lehnert	1	0	0
Cyatheaceae	Cyathea	*Cyathea* aff. *patens* H.Karst.	0	1	0
Dicksoniaceae	Dicksonia	*Dicksonia sellowiana* (C.Presl) Hook.	1	0	1
Escalloniaceae	Escallonia	*Escallonia pendula* (Ruiz & Pav.) Pers.	0	0	1
Euphorbiaceae	Alchornea	*Alchornea brittonii* Secco	1	0	0
Euphorbiaceae	Alchornea	*Alchornea glandulosa* Poepp.	0	0	1
Euphorbiaceae	Alchornea	*Alchornea triplinervia* (Spreng.) Müll. Arg.	1	0	1
Euphorbiaceae	Alchornea	*Alchornea grandiflora* Müll.Arg.	0	1	0
Euphorbiaceae	Tetrorchidium	*Tetrorchidium rubrivenium* Poepp. & Endl.	1	0	0
Fabaceae	Erythrina	*Erythrina ulei* Harms	1	0	0
Fabaceae	Inga	*Inga setosa* G. Don	0	0	1
Gentianaceae	Macrocarpaea	*Macrocarpaea* cf. *Kuelap* J.R. Grant	0	1	0
Hypericaceae	Vismia	*Vismia* sp.1	1	0	0
Indet.	Indet.	Morphospecie 1	1	0	0
Indet.	Indet.	Morphospecie 2	0	0	1
Lauraceae	Aiouea	*Aiouea montana* (Sw.) R. Rohde	1	0	1
Lauraceae	Aniba	*Aniba coto* (Rusby) Kosterm.	0	1	0
Lauraceae	Aniba	*Aniba muca* (Ruiz & Pav.) Mez.	1	0	0
Lauraceae	Aniba	*Aniba* sp.1	1	0	0
Lauraceae	Beilschmiedia	*Beilschmiedia* sp.1	0	0	1
Lauraceae	Beilschmiedia	*Beilschmiedia tovarensis* (Klotzsch & H.Karst. ex Meisn.) Sachiko Nishida	1	0	1
Lauraceae	Licaria	*Licaria triandra* (Sw.) Kosterm.	0	0	1
Lauraceae	Nectandra	*Nectandra cissiflora* Nees	1	0	1
Lauraceae	Nectandra	*Nectandra cuspidata* Ness &Mart. ex Nees	1	0	0
Lauraceae	Nectandra	*Nectandra discolor* (Kunth) Nees	0	1	0
Lauraceae	Nectandra	*Nectandra oppositifolia* Nees & Mart. ex Nees	1	0	0
Lauraceae	Nectandra	*Nectandra* aff. *reflexa* Rohwer	1	0	0
Lauraceae	Ocotea	*Ocotea aciphylla* (Nees) Mez	0	0	1
Lauraceae	Ocotea	*Ocotea argyrophylla* Ducke	0	1	0
Lauraceae	Ocotea	*Ocotea* aff. *balanocarpa* (Ruiz & Pav.) Mez	0	0	1
Lauraceae	Ocotea	*Ocotea benthamiana* Mez	0	1	0
Lauraceae	Ocotea	*Ocotea bofo* Kunth	0	1	1
Lauraceae	Ocotea	*Ocotea* aff. *insularis* (Meins.) Mez	0	0	1
Lauraceae	Ocotea	*Ocotea olivacea* A. C. Sm.	1	0	0
Lauraceae	Ocotea	*Ocotea* aff. *olivacea* A. C. Sm.	1	0	0
Lauraceae	Ocotea	*Ocotea* sp.1	0	1	0
Lauraceae	Ocotea	*Ocotea* sp.2	0	1	0
Lauraceae	Persea	*Persea* sp.1	1	0	0
Lauraceae	Persea	*Persea brevipes* Meisn.	0	1	0
Lauraceae	Persea	*Persea caerulea* (Ruiz & Pav.) Mez.	1	0	1
Malpighiaceae	Byrsonima	*Byrsonima garcibarrigae* Cuatrec.	0	0	1
Melastomataceae	Graffenrieda	*Graffenrieda emarginata* Triana	0	1	0
Melastomataceae	Miconia	*Miconia aureoides* Cogn.	1	0	0
Melastomataceae	Miconia	*Miconia poeppigii* Triana	1	0	0
Melastomataceae	Miconia	*Miconia sessiliflora* (Naudin) Michelang.	0	0	1
Melastomataceae	Miconia	*Miconia* sp.1	1	1	1
Melastomataceae	Miconia	*Miconia* sp.2	0	1	0
Meliaceae	Cedrela	*Cedrela montana* Moritz ex Turcz.	1	0	0
Meliaceae	Guarea	*Guarea kunthiana* A. Juss.	1	0	0
Meliaceae	Ruagea	*Ruagea glabra* Triana & Planch.	1	0	0
Meliaceae	Trichilia	*Trichilia tomentosa* Kunth	1	0	0
Monimiaceae	Mollinedia	*Mollinedia lanceolata* Ruiz & Pav.	1	0	0
Moraceae	Ficus	*Ficus citrifolia* Mill.	0	0	1
Moraceae	Ficus	*Ficus cuatrecasasiana* Dugand	1	0	0
Moraceae	Ficus	*Ficus gigantosyce* Dugand	1	0	0
Moraceae	Ficus	*Ficus maximoides* C. C. Berg	1	0	0
Moraceae	Morus	*Paratrophis insignis* (Bureau) E.M.Gardner	1	0	0
Myrtaceae	Myrcia	*Myrcia aliena* McVaugh	0	1	0
Myrtaceae	Eugenia	*Eugenia* sp.1	0	1	0
Myrtaceae	Eugenia	*Eugenia* sp.2	0	1	0
Myrtaceae	Myrcia	*Myrcia densiflora* (Poepp. Ex O.Berg) A.R. Lourenço E.Lucas	0	0	1
Myrtaceae	Myrcia	*Myrcia megapaniculata* A.R.Lourenço & Parra-Os	0	0	1
Myrtaceae	Myrcia	*Myrcia simulata* (McVaugh) A.R.Lourenço & Parra-Os	0	0	1
Myrtaceae	Myrcia	*Myrcia* sp.1	0	1	0
Myrtaceae	Myrciaria	*Myrciaria* sp.1	0	1	0
Myrtaceae	Myrciaria	*Myrciaria tenella* (DC.) O.Berg	0	1	0
Pentaphylacaceae	Ternstroemia	*Ternstroemia jelskii* (Szyszyl.) Melch.	0	1	0
Pentaphylacaceae	Ternstroemia	*Ternstroemia* sp.1	0	1	0
Phyllanthaceae	Hieronyma	*Hieronyma asperifolia* Pax & K. Hoffm.	1	0	1
Phyllanthaceae	Hieronyma	*Hieronyma* sp.1	1	0	1
Piperaceae	Piper	*Piper heterophyllum* Ruiz & Pav.	0	0	1
Piperaceae	Piper	*Piper obliquum* Ruiz & Pav.	0	0	1
Podocarpaceae	Podocarpus	*Podocarpus oleifolius* D.Don	0	1	1
Primulaceae	Myrsine	*Myrsine coriacea* (Sw.) R.Br. Ex Roem. & Schult.	1	1	0
Primulaceae	Myrsine	*Myrsine latifolia* (Ruiz & Pav.) Spreng.	1	1	0
Rosaceae	Prunus	*Prunus guanaiensis* Rusby	1	0	0
Rosaceae	Prunus	*Prunus integrifolia* (C.Presl) Walp.	0	0	1
Rubiaceae	Chimarrhis	*Chimarrhis* sp.1	0	0	1
Rubiaceae	Cinchona	*Cinchona parabolica* Pav.	0	1	0
Rubiaceae	Holtonia	*Holtonia microcarpa* (Ruiz & Pav.) C.M.Taylor	1	0	0
Rubiaceae	Faramea	*Faramea jasminoides* (Kunth) DC.	0	1	1
Rubiaceae	Tournefortiopsis	*Tournefortiopsis hirsuta* (Ruiz & Pav.) Pers.	1	0	0
Rubiaceae	Psychotria	*Psychotria* sp.1	0	0	1
Rubiaceae	Palicourea	*Palicourea sulphurea* (Ruiz & Pav.) DC.	1	0	0
Rubiaceae	Indet.	morphospecie 1	0	0	1
Rutaceae	Amyris	*Amyris* cf. *pinnata* Kunth	0	1	0
Sabiaceae	Meliosma	*Meliosma frondosa* Cuatrc. & Idrobo	1	0	0
Sabiaceae	Meliosma	*Meliosma* aff. *sirensis* A.H. Gentry	0	1	0
Sabiaceae	Meliosma	*Meliosma* sp.1	1	0	0
Salicaceae	Irenodendron	*Irenodendron ovalifolium* (J.F.Macbr.) M.H.Alford & Dement	0	0	1
Sapindaceae	Allophylus	*Allophylus floribundus* Radlk.	1	0	0
Sapindaceae	Cupania	*Cupania cinerea* Poepp. & Endl.	1	0	0
Siparunaceae	Siparuna	*Siparuna tomentosa* (Ruiz & Pav.) A.DC.	0	1	0
Solanaceae	Cestrum	*Cestrum* sp.1	1	0	0
Solanaceae	Solanum	*Solanum* sp.1	1	0	0
Styracaceae	Styrax	*Styrax cordatus* A. DC.	0	1	0
Styracaceae	Styrax	*Styrax pavonii* A. DC.	1	0	0
Styracaceae	Styrax	*Styrax peruvianus* Zahlbr.	0	1	1
Symplocaceae	Symplocos	*Symplocos fuliginosa* B. Ståhl	1	0	1
Symplocaceae	Symplocos	*Symplocos ovata* B. Ståhl	0	1	0
Symplocaceae	Symplocos	*Symplocos peruviana* (Szyszyt.) Brand	1	0	0
Theaceae	Gordonia	*Gordonia fruticosa* (Schrad.) H. Keng	0	1	0
Urticaceae	Cecropia	*Cecropia* sp.1	1	0	0
Urticaceae	Cecropia	*Cecropia telenitida* Cuatrec.	0	0	1
Urticaceae	Myriocarpa	*Myriocarpa stipitata* Benth.	1	0	0
Urticaceae	Pouzolzia	*Pouzolzia obliqua* (Wedd.) Wedd	0	0	1
Verbenaceae	Citharexylum	*Citharexylum* aff. *subflavescens* S. F. Blake	1	0	0
Viburnaceae	Viburnum	*Viburnum wurdackii* T. R. Dudley	1	0	0
